# Preventive Effects of Different Aerobic Exercise Intensities on the Decline of Cognitive Function in High-Fat Diet-Induced Obese Growing Mice

**DOI:** 10.3390/medicina56070331

**Published:** 2020-07-02

**Authors:** Ju Yong Bae

**Affiliations:** Laboratory of Exercise Biochemistry, Department of Physical Education, College of Arts and Physical Education, Dong-A University, 37 Nakdong-daero 550 beon-gil, Hadan-dong, Saha-gu, Busan 604-714, Korea; kosa99@dau.ac.kr; Tel.: +82-51-200-7836; Fax: +82-51-200-7805

**Keywords:** treadmill training, obesity, neurotrophins, NGF, BDNF, NT-3

## Abstract

*Background and Objectives:* The purpose of this study was to elucidate the effects of different exercise intensities in preventing the decline of cognitive function and lipolysis associated with a high-fat diet-induced obesity in growing mice. *Material and Methods:* Forty male C57BL/6 mice, aged 4 weeks, were divided into the normal diet (CO, *n* = 10) and high-fat diet (HF, *n* = 30) groups to induce obesity for 8 weeks. Subsequently, the HF group was subdivided equally into the HF, HF + low-intensity training (HFLT), and HF + high-intensity training (HFHT) groups, and mice were subjected to treadmill training for 8 weeks. *Results:* Following the 8-week training intervention, body weight and fat mass were significantly lower in the training groups than in the HF group (*p* < 0.05). Adipose triglyceride lipase (ATGL), hormone-sensitive lipase (HSL), and monoglyceride lipase levels were significantly higher in the training groups than in the HF group (*p* < 0.05), and the ATGL and HSL levels were significantly higher in the HFHT group than in the HFLT group (*p* < 0.05). The Y-maze test showed that the training groups had a higher number of total entries and percent alternation than the HF group (*p* < 0.05). Hippocampal nerve growth factor, brain-derived neurotrophic factor, and neurotrophin-3 levels were significantly higher in the training group than in the HF group (*p* < 0.05). However, there was no significant difference according to the exercise intensity among the groups. *Conclusions:* The results of this study suggested that low-intensity exercise is as effective as a high-intensity exercise in preventing the decline of cognitive function and lipolysis, and far more effective in terms of an expected efficiency of workload and prevention of side effects.

## 1. Introduction

An imbalance of energy metabolism occurs due to high-fat diet (HFD)-induced obesity, leading to adult diseases such as metabolic syndrome, cardiovascular diseases, and type 2 diabetes [1]. Along with metabolic and cardiovascular diseases, obesity has adverse cognitive outcomes [2]. The prevalence of obesity has increased consistently [3], especially childhood obesity, which has increased at a high rate in the past three decades [4]. Obesity in the developmental periods can be particularly problematic because it is crucial to the maturation of the hippocampus [5].

The hippocampus is a region of the brain associated with cognitive functions, particularly with learning and memory. This part of the brain plays a critical role in the creation of new memories as well as in the processing of declarative and spatial memory [6]. Aging is a well-known and powerful promoter of cognitive decline, and chronic stress [7], oxidative stress [8], and increased pro-inflammatory cytokine expression in the hippocampus [6] have similar effects independent of aging.

On the other hand, neurogenesis and neural development and maintenance by the upregulation of neurotrophic factors are associated with improved cognitive function. Neurotrophic factors such as nerve growth factor (NGF), brain-derived neurotrophic factor (BDNF), and neurotrophin-3 (NT-3) are secreted proteins that promote neuronal cell differentiation, survival, and neurite outgrowth [9]. NGF is a fully characterized molecule that is present in the central nervous system (CNS), where it serves a trophic function in the differentiation and maintenance of basal forebrain cholinergic neurons [10]. BDNF is mostly expressed in the CNS and has a critical role in hippocampal neurogenesis, experience-dependent neuroplasticity, neuronal shaping, and survival [11]. NT-3 was discovered in 1990 as the third member of the neurotrophin family [12], and it acts as a contributor to the survival of neurons of the hippocampus, sympathetic ganglia, and dorsal root ganglia [13].

Evidence that HFD induces cognitive impairment, but that exercise improves cognitive function, are well established in both animal and human studies. A previous study reported that obesity caused by HFD induced insulin resistance and memory dysfunction; however, physical exercise improved mitochondrial function and inhibited apoptosis in the hippocampus [14]. Moreover, treadmill running improved HFD-induced cognitive impairment by improving the key molecules involved in Alzheimer’s disease pathology [15]. Even in the case of human studies, physical exercise positively affects cognitive function in healthy subjects [16], as well as patients with Alzheimer’s disease [17] and Parkinson’s disease [18]. Regular exercise is one of the most effective ways to alleviate induced obesity by increasing lipolysis [19] and additionally has the advantage of inducing an increase in neurotrophins, which may lead to improved cognitive function [20]. Moreover, exercise improves learning and memories, which is accompanied by increased hippocampal cell proliferation and survival [21].

In an interesting previous study, exposure to HFD for 2 months in adult mice did not affect the hippocampal function, but exposure to HFD in adolescent mice abolished relational memory flexibility, discrimination learning, and decreased neurogenesis [22]. As a result of previous studies comparing the effects of exercising during adulthood versus adolescence, it was found that exercise during adolescence had a greater object recognition memory and that memory was maintained longer than exercise during adulthood [23]. Despite the sensitivity to HFD and physical exercise in adolescence, most studies have focused on cognitive function in adults.

The effects of exercise can vary depending on the different intensities of the exercise. Traditionally, a moderate-intensity exercise in children has been the recommended exercise to improve body composition and health-related factors [24]. To induce a similar effect of moderate-intensity exercise, the training duration of low-intensity exercise should be sufficiently long. Although high-intensity training may improve physiological adaptations and may induce greater health benefits because of a high amount of total workload [25], high-intensity training may induce greater fatigue [26]. Regular exercise seems to induce improvement in cognitive function with the alleviation of obesity, but the effects based on exercise intensity in the adolescent period remain unclear.

Therefore, the present study aimed to elucidate the effects of different exercise intensities in preventing the decline of cognitive function and lipolysis associated with HFD-induced obesity in growing mice.

## 2. Materials and Methods

### 2.1. Animals and Obesity Induction

This study included forty 4-week-old, male C57BL/6 mice. Four mice were housed per cage in the Dong-A University College of Medicine Animal Laboratory. The laboratory conditions were controlled for relative humidity (55 ± 5%), temperature (22 ± 2 °C), and light (12-h dark–light cycle; 07:00 light on, 19:00 light off). Animals were randomly divided into two groups: normal diet (CO, *n* = 10) and HFD (HF, *n* = 30). The HF group was fed a 60% fat chow (60% lipid, 20% carbohydrate, and 20% protein; D-12492, Research Diets, Inc., Brunswick, NJ, USA) to induce obesity, whereas the CO group was fed a standard chow (6.3% lipid, 69.4% carbohydrate, and 24.3% protein; DongA 1 Corporation, Dangjin-gun, Choongnam, Korea) for 8 weeks. The animal experiments were approved by the Dong-A University Medical School Institutional Animal Care and Use Committee (DIACUC-approval-17-9, date of approval: 30 June 2017), and all procedures were conducted in accordance with the committee guidelines.

### 2.2. Exercise Intervention

After 8 weeks of the obesity induction period, the mice in the HF group were further subdivided randomly into the HF (*n* = 10), HF + low-intensity training (HFLT, *n* = 10), and HF + high-intensity training (HFHT, *n* = 10) groups. Animals in the training groups exercised on the animal treadmill five times a week for eight weeks while maintaining HFD consumption.

In the first week, mice were exposed to the treadmill for 5 min a day at a speed of 5 m/min for exercise adaptation, and the exposure time was gradually increased to 20 min. On the fifth day of exercise adaptation, the incremental load test was conducted to determine the treadmill exercise speed of the HFLT and HFHT groups by referring to the previous study [27]. Briefly, after 5 min warm-up at 5 m/min, the speed of the treadmill was increased by 3 m/min every 3 min at 0% grade until exhaustion. As a result of the incremental load test of the exercise group, the average maximum speed was 19.1 ± 0.44 m/min, and the average exercise duration was 20.02 ± 0.34 min. The speeds in the HFLT (40~45%) and HFHT (75~80%) were relatively determined by corresponding to the maximal speed of training groups. The mice in the exercise groups performed a total of 50 min of exercise a day, including 10 min of warm-up and 10 min of cool-down exercise, and the specific exercise protocol is shown in Table 1. To expose a similar stress condition with training groups, CO and HF mice that did not perform the exercise were placed next to the treadmill while the training groups were conducting treadmill training.

### 2.3. Cognitive Function Test

To evaluate cognitive function, spontaneous alternation was tested in a Y-maze just before dissection [28]. Spontaneous alternation in a Y-maze can measure spatial memory by allowing mice to explore all three arms of the maze freely [29] with very little stress [30]. A mouse with a good memory remembers the arm of the maze that has already been visited and tends to get into the less-visited arm [31]. The Y-maze was designed with three arms of 40 cm length, 13.5 cm height, and 4 cm width, and the test was performed under bright room conditions. Each mouse was placed at the end of the arm and allowed to move through the maze for 5 min. Arm entries were scored by a trained observer who did not know the treatment group. Alternation was defined as consecutive entries into the three different arms for overlapping triplet sets. Percent alternation was calculated by the following formula: % alternation = (number of entries in the other direction/(total number of entries – 2) × 100).

### 2.4. Protein Level Analysis

To exclude the temporary training effects, tissue sampling was conducted 48 h after the completion of the last exercise. Food was withdrawn from the mouse cages 12 h before sacrifice. The epididymal adipose and hippocampus samples were excised after complete anesthesia using ethyl ether. The samples were immediately frozen in liquid nitrogen and stored at −80 °C. As previously described [32], the tissue was homogenized in 200 μL of radioimmunoprecipitation assay buffer to extract protein from the adipose and hippocampus samples, and then centrifuged at 14,000 rpm for 30 min. The protein concentration was measured using the Bicinchoninic Acid protein assay kit (Catalog number: 23225, Thermo Scientific, Waltham, MA, USA). Samples of equal protein content were separated by SDS-PAGE and then transferred to a polyvinylidene difluoride membrane. The membrane was blocked with 3% skimmed milk in phosphate-buffered saline, and incubated at 4 °C overnight with primary antibodies against adipose triglyceride lipase (ATGL; sc-67355), hormone-sensitive lipase (HSL; sc-25843), monoglyceride lipase (MGL; sc-398942), nerve growth factor (NGF; sc-365944), brain-derived neurotrophic factor (BDNF; sc-65514), and neurotrophin-3 (NT-3; sc-547) (all antibodies were purchased from Santa Cruz Biotechnology, Dallas, TX, USA). The membrane was incubated with goat anti-mouse or anti-rabbit immunoglobulin-G conjugated secondary antibody for 1 h at room temperature. The protein bands were developed using the ImageQuantTM LAS-4000 system (GE Healthcare, Uppsala, Sweden) through an enhanced chemiluminescence solution (Amersham Pharmacia Biotech, Marlborough, USA).

### 2.5. Statistical Analysis

All the statistical analyses were performed using SPSS 22.0 (SPSS Inc., Chicago, IL, USA), and the results were expressed as mean ± standard error. Shapiro–Wilk tests were performed to confirm that all data were distributed normally within each group. Two-way repeated measures ANOVA was used to analyze the changes in body weight by high-fat diet and exercise intervention, and one-way ANOVA followed by a Duncan post hoc test to compare the differences between groups after exercise intervention. Values of *p* < 0.05 were considered statistically significant.

## 3. Results

### 3.1. HFD Caused Obesity and Increased Fat Mass, but That Was Prevented by Regular Exercise

**Figure 1:**
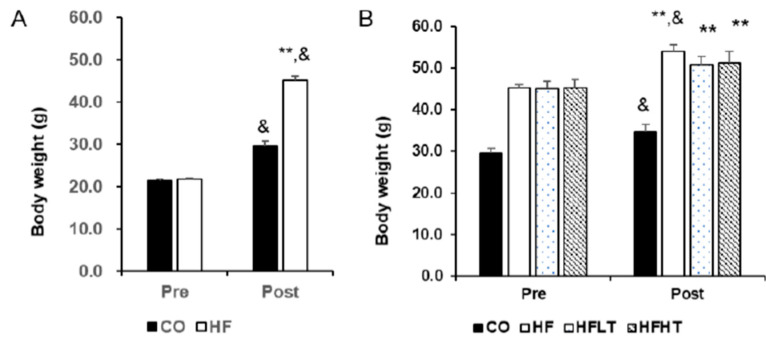
Changes of body weight after intervention. Body weight after 8 weeks of high-fat diet (**A**) and 8 weeks of training (**B**) are presented. Data are expressed as mean ± SE. CO, normal-diet group; HF, high-fat diet group; HFLT, high-fat diet + low-intensity training group; HFHT, high-fat diet + high-intensity training group. &, versus before, *p* < 0.05; ** versus CO group, *p* < 0.001.

**Figure 2:**
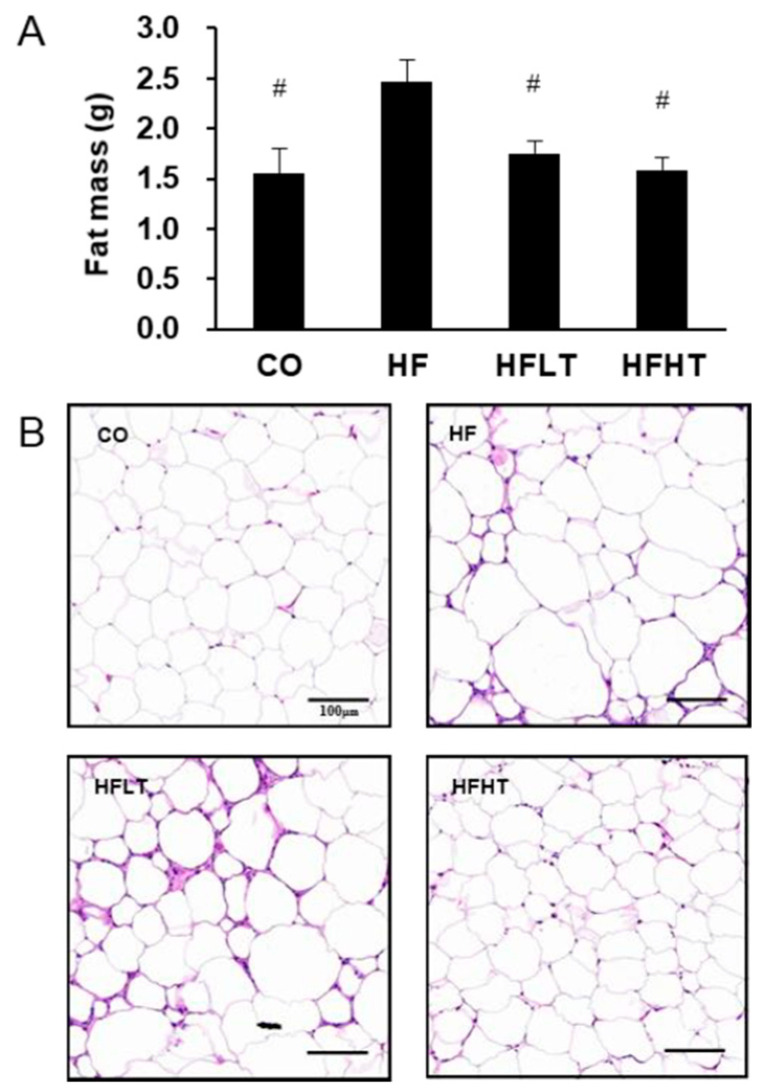
Changes of adipose tissue after intervention. Changes in fat mass (**A**) and lipid droplet (**B**) are presented. Data are expressed as mean ± SE. # versus HF group, *p* < 0.05. Scale bar = 100 μm. CO, normal-diet group; HF, high-fat diet group; HFLT, high-fat diet + low-intensity training group; HFHT, high-fat diet + high-intensity training group.

Changes in body weight before and after the intervention are shown in Figure 1. Following 8 weeks of HFD (Figure 1A), repeated ANOVA measures demonstrated a significant difference across time by group interaction (F = 220.014, *p* = 0.000). Post hoc analysis revealed that obesity was induced based on a significant increase in body weight in the HF group after 8 weeks of HFD (t = 45.267, *p* = 0.000). Following 8 weeks of exercise intervention (Figure 1B), repeated ANOVA measures also demonstrated a significant difference across time by group interaction (F = 3.111, *p* = 0.038). Although the body weight of training group participants did not increase, there was an increase in the CO (t = 5.989, *p* = 0.000) and HF group (t = 12.928, *p* = 0.000) compared with before exercise intervention (Figure 1B). After 8 weeks of exercise intervention, fat mass was significantly higher in the HF group than in the other groups (F = 5.237, *p* < 0.05) (Figure 2A). The size of the lipid droplet of the HF group was larger than that of the CO group. However, the LD size of the training groups decreased, and even the lipid droplet of the HFHT group was similar to that of the CO group (Figure 2B).

### 3.2. Lipolytic Enzymes in Adipose Tissue Increased in Training Groups, and High-Intensity Training Was More Effective than That of Low-Intensity

**Figure 3:**
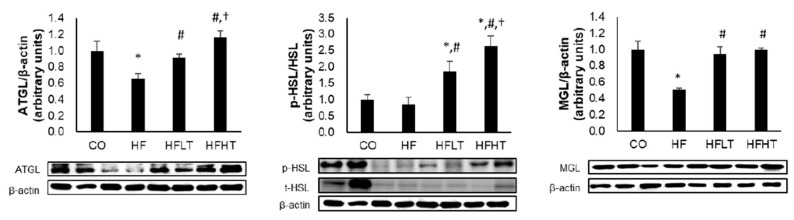
Changes of lipolysis enzyme in adipose tissue after intervention. Data are expressed as mean ± SE. * versus CO group, *p* < 0.05; # versus HF group, *p* < 0.05; † versus HFLT group, *p* < 0.05. ATGL, adipose triglyceride lipase; p- and t-HSL, phospho- and total-hormone sensitive lipase; MGL, monoglyceride lipase; CO, normal-diet group; HF, high-fat diet group; HFLT, high-fat diet + low-intensity training group; HFHT, high-fat diet + high-intensity training group.

Following 8 weeks of exercise intervention, lipolytic enzymes were significantly different between groups for adipose triglyceride lipase (ATGL) (F = 10.628, *p* = 0.000), hormone-sensitive lipase (p-HSL/t-HSL) (F = 17.936, *p* = 0.000), and monoglyceride lipase (MGL) levels (F = 17.129, *p* = 0.000). The ATGL and MGL levels were significantly lower in the HF group than in the CO group (*p* < 0.05) (Figure 3). The ATGL, p-HSL/t-HSL, and MGL levels were significantly higher in training groups than in the HF group (*p* < 0.05), and ATGL and p-HSL/t-HSL levels were significantly higher in the HFHT group than in the HFLT group after 8 weeks of training intervention (*p* < 0.05) (Figure 3).

### 3.3. Cognitive Function and Neurotrophic Factors Were Improved by Regular Exercise Regardless of the Exercise Intensity

**Figure 4:**
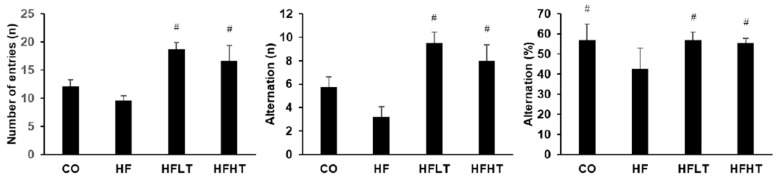
Results of Y-maze test after intervention. Data are expressed as mean ± SE. # versus HF group, *p* < 0.05. CO, normal-diet group; HF, high-fat diet group; HFLT, high-fat diet + low-intensity training group; HFHT, high-fat diet + high-intensity training group.

After 8 weeks of the training intervention, the Y-maze test was conducted to analyze spatial cognitive function. Following 8 weeks of exercise intervention, cognitive function was a significant difference between groups for the number of entries (F = 8.013, *p* = 0.000), alternations (F = 8.744, *p* = 0.000), and % alternation (F = 3.197, *p* = 0.000). The numbers of entries and alternations were significantly higher in the training groups than in the HF group (*p* < 0.05). The % alternation was significantly lower in the HF group than in all other groups (*p* < 0.05) (Figure 4).

**Figure 5:**
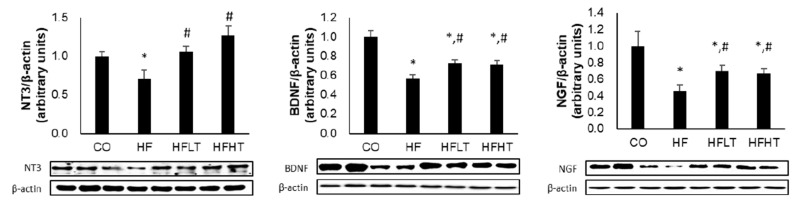
Changes of neurotrophic factors in hippocampus after intervention. Data are expressed as mean ± SE. * versus CO group, *p* < 0.05; # versus HF group, *p* < 0.05. NT-3, neurotrophin-3; BDNF, brain-derived neurotrophic factor; NGF, nerve growth factor; CO, normal-diet group; HF, high-fat diet group; HFLT, high-fat diet + low-intensity training group; HFHT, high-fat diet + high-intensity training group.

Following 8 weeks of exercise intervention, neurotrophic factors were significant differences between groups for the NGF (F = 7.360, *p* = 0.001), BDNF (F = 25.687, *p* = 0.000), and NT-3 (F = 8.752, *p* = 0.000). Hippocampal NGF, BDNF, and NT-3 levels were significantly lower in the HF group than in the CO group after the intervention (*p* < 0.05). However, hippocampal NGF, BDNF, and NT-3 levels were significantly higher in the training groups than in the HF group (*p* < 0.05) (Figure 5). However, cognitive function and neurotrophic factors were not significant differences between exercise intensities.

## 4. Discussion

The results of this study showed that, although HFD intake reduces lipolytic enzymes with an increase in body weight and fat mass, regular exercise increased p-HSL, ATGL, and MGL protein levels with a decrease in body weight and fat mass. Moreover, there was no difference in body weight and fat mass based on the exercise intensity, but the HFHT group showed an increase in the lipolysis-related factors compared to the HFLT group. Concerning cognitive function, training groups showed a significant increase in hippocampal NGF, BDNF, and NT-3 protein levels and spatial cognitive abilities, but there was no significant difference in the different exercise intensities.

The overall prevalence of obesity is steadily increasing globally, which is becoming more severe [33]. Unhealthy eating habits are a powerful cause of obesity; particularly, the consumption of chronic HFD leads to metabolic imbalances. Exercise is one of the most effective ways to alleviate obesity by promoting lipolysis without side effects [34]. A previous study also suggested that low-calorie dietary conversion with regular exercise has synergistic effects on reducing weight and fat mass [35]. However, we confirmed that regular exercise was effective in reducing fat mass but not effective in weight reduction in this study. In fact, neither did the body weight of the exercise group decrease nor did the body weight increase significantly before exercise. It is important to note that the mice used in the present study were in their growth phases where they would continue to gain weight physiologically, and the weight gain was inhibited by regular exercise even though they had not been switched to a low-fat diet. 

Excessive calories from the consumption of HFD are stored in the form of triacylglycerides (TAGs) in lipid droplets [32]. TAG is hydrolyzed sequentially to diacylglycerols and monoacylglycerols, and the latter is finally hydrolyzed to fatty acids and glycerol. ATGL, HSL, and MGL act in turn in each step of decomposition of lipolysis [36]. We analyzed the effects of different intensities of exercise on these lipolytic enzymes and fat mass. Regular exercise induced an increase in lipolytic enzymes and a decrease in fat mass. Furthermore, there were no differences in body weight and fat mass based on the exercise intensity, but lipolytic enzyme levels increased in the high-intensity exercise group compared to those in the low-intensity group. Exercise intensity is the most relevant factor in fat oxidation. Although fat oxidation is the highest at low-to-moderate intensities, well-trained athletes have their highest fat oxidation at the ventilatory threshold [37]. Therefore, the amount of fat oxidation is influenced by various factors, such as the age and aerobic fitness of the subject. A study including young men subjected to different intensities (45% and 65% of VO2 peak) of aerobic exercise with the same relative workload showed that the plasma free fatty acids flux increased during exercise, but total fat oxidation and whole-body lipolysis were unaffected [38]. The total workload was not measured in this study, but the two groups performed different intensity exercises for the same time; thus, the high-intensity exercise group performed a relatively larger amount of work. This is believed to contribute to an increase in the lipolytic enzymes of the high-intensity exercise group, but it did not cause a decrease in body weight or body fat. Therefore, further measurement of muscle mass and dietary intake will be necessary for a detailed analysis of exercise intensity on body weight and fat mass.

Obesity due to HFD not only increases insulin resistance, fat accumulation, and adipose tissue inflammation but also negatively affects brain function by impairing hippocampal function [39]. On the other hand, there is convergent evidence that exercise improves brain function and cognitive function throughout the mammalian lifespan. Aerobic exercise enhances the production of neuroprotective trophic factors and promotes neuronal survival [40], and cholinergic alterations induced by spatial learning and physical activity were observed in the hippocampus [41]. Moreover, aerobic exercise training improved memory function by restoring the hippocampal volume reduction at the end of adulthood, and exercise improved the hippocampus-dependent spatial memory in the Morris water maze, Y-maze, and radial-arm maze tests [42]. The role of regular exercise in improving memory function is well documented; however, most studies have focused on old adults and healthy subjects [41,42]. Additionally, differences in the effects of exercise intensity on HFD-induced obese growing mice have not been elucidated. Thus, we hypothesized that although obesity at the growing period harms cognitive function, regular exercise may prevent deterioration of cognitive function and the effect could vary depending on the exercise intensity. Similar to previous studies, we confirmed that regular aerobic exercise induced an increase in the level of hippocampal neurotrophins and improvement of the spatial cognitive function in obese mice, but the difference based on the exercise intensity was not confirmed. These results indicate that continuous performing of exercise is more important, regardless of exercise intensity to improve cognitive function.

High-intensity exercise induces greater health benefits than low-intensity exercise when performing the same duration of exercise due to a large amount of total workload [25]. On the other hand, hippocampal synaptic plasticity and spatial learning behavior could be impaired by high-intensity exercise [43], and excessively intense exercise could induce brain dysfunction resulting from fatigue and stress generation [44]. In this study, the additional benefits of high-intensity exercise for cognitive function were not identified; therefore, low-intensity exercise can be recommended for growing children in terms of avoiding side effects.

## 5. Conclusions

To summarize, even in the case of obesity due to HFD at a growing period, regular aerobic exercise is effective in maintaining body weight and fat mass, as well as improvement of cognitive function. Although high-intensity exercise induced an increase in lipolytic enzymes, further benefits could not be confirmed despite the fact that the HFHT group performed a large amount of workload compared with the low-intensity exercise group. Therefore, this study suggests that low-intensity exercise is as effective as the high-intensity exercise to prevent the decline of cognitive function and promote lipolysis and is far more effective in terms of an expected efficiency of workload and creating less fatigue.

## Figures and Tables

**Table 1:** Training protocol.

Period (Week)		Speed (m/min)		Time (Min)	Frequency (Day/Week)	Grade (°)
		Low Intensity	High Intensity			
1	Adaptation	5	5	5–20	5	0
2~8	Exercise	5	5	10	5	0
		8	14	30		
		5	5	10		

## References

[1] Langin D. (2006). Adipose tissue lipolysis as a metabolic pathway to define pharmacological strategies against obesity and the metabolic syndrome. Pharmacol. Res..

[2] Boitard C., Cavaroc A., Sauvant J., Aubert A., Castanon N., Layé S., Ferreira G. (2014). Impairment of hippocampal-dependent memory induced by juvenile high-fat diet intake is associated with enhanced hippocampal inflammation in rats. Brain. Behav. Immun..

[3] Stevens G.A., Singh Lu Y., Danaei G., Lin J.K., Finucane M.M., Bahalim A.N., McIntire R.K., Gutierrez H.R., Cowan M., Paciorek C.J. (2012). Global Burden of Metabolic Risk Factors of Chronic Diseases Collaborating Group (Body Mass Index). National, regional, and global trends in adult overweight and obesity prevalences. Popul. Health Metr..

[4] Güngör N.K. (2014). Overweight and obesity in children and adolescents. J. Clin. Res. Pediatr. Endocrinol..

[5] Spear L.P. (2000). The adolescent brain and age-related behavioral manifestations. Neurosci. Biobehav. Rev..

[6] Biegler R., McGregor A., Krebs J.R., Healy S.D. (2001). A larger hippocampus is associated with longer-lasting spatial memory. Proc. Natl. Acad. USA..

[7] Phillips C. (2017). Lifestyle Modulators of Neuroplasticity: How Physical Activity, Mental Engagement, and Diet Promote Cognitive Health during Aging. Neural. Plast..

[8] Kruk-Slomka M., Boguszewska-Czubara A., Slomka T., Budzynska B., Biala G. (2016). Correlations between the Memory-Related Behavior and the Level of Oxidative Stress Biomarkers in the Mice Brain, Provoked by an Acute Administration of CB Receptor Ligands. Neural. Plast..

[9] Skaper S.D. (2018). Neurotrophic Factors: An Overview. Methods Mol. Biol..

[10] Dreyfus C.F. (1989). Effects of nerve growth factor on cholinergic brain neurons. Trends Pharmacol. Sci..

[11] Deinhardt K., Chao M.V. (2014). Shaping neurons: Long and short range effects of mature and proBDNF signalling upon neuronal structure. Neuropharmacology.

[12] Rosenthal A., Goeddel D.V., Nguyen T., Lewis M., Shih A., Laramee G.R., Nikolics K., Winslow J.W. (1990). Primary structure and biological activity of a novel human neurotrophic factor. Neuron.

[13] Kucera J., Fan G., Jaenisch R., Linnarsson S., Ernfors P. (1995). Dependence of developing group la afferents on neurotrophin-3. J. Comp. Neurol..

[14] Park H.S., Cho H.S., Kim T.W. (2018). Physical exercise promotes memory capability by enhancing hippocampal mitochondrial functions and inhibiting apoptosis in obesity-induced insulin resistance by high fat diet. Metab. Brain Dis..

[15] Cheng J., Chen L., Han S., Qin L., Chen N., Wan Z. (2016). Treadmill Running and Rutin Reverse High Fat Diet Induced Cognitive Impairment in Diet Induced Obese Mice. J. Nutr. Health Aging..

[16] Coetsee C., Terblanche E. (2017). The effect of three different exercise training modalities on cognitive and physical function in a healthy older population. Eu.r Rev. Aging Phys. Act..

[17] Cui M.Y., Lin Y., Sheng J.Y., Zhang X., Cui R.J. (2018). Exercise Intervention Associated with Cognitive Improvement in Alzheimer’s Disease. Neural. Plast..

[18] Da Silva F.C., Iop R.D.R., de Oliveira L.C., Boll A.M., de Alvarenga J.G.S., Gutierres Filho P.J.B., de Melo L.M.A.B., Xavier A.J., da Silva R. (2018). Effects of physical exercise programs on cognitive function in Parkinson’s disease patients: A systematic review of randomized controlled trials of the last 10 years. PLoS ONE.

[19] van Praag H., Shubert T., Zhao C., Gage F.H. (2005). Exercise enhances learning and hippocampal neurogenesis in aged mice. J. Neurosci..

[20] Birch A.M., McGarry N.B., Kelly A.M. (2013). Short-term environmental enrichment, in the absence of exercise, improves memory, and increases NGF concentration, early neuronal survival, and synaptogenesis in the dentate gyrus in a time-dependent manner. Hippocampus.

[21] Woo J., Shin K.O., Park S.Y., Jang K.S., Kang S. (2013). Effects of exercise and diet change on cognition function and synaptic plasticity in high fat diet induced obese rats. Lipids Health Dis..

[22] Boitard C., Etchamendy N., Sauvant J., Aubert A., Tronel S., Marighetto A., Layé S., Ferreira G. (2012). Juvenile, but not adult exposure to high-fat diet impairs relational memory and hippocampal neurogenesis in mice. Hippocampus.

[23] Hopkins M.E., Nitecki R., Bucci D.J. (2011). Physical exercise during adolescence versus adulthood: Differential effects on object recognition memory and brain-derived neurotrophic factor levels. Neuroscience.

[24] Aucouturier J., Baker J.S., Duché P. (2008). Fat and carbohydrate metabolism during submaximal exercise in children. Sport Med..

[25] Boutcher S.H. (2011). High-intensity intermittent exercise and fat loss. J. Obes..

[26] Lattier G., Millet G.Y., Martin A., Martin V. (2004). Fatigue and recovery after high-intensity exercise part I: Neuromuscular fatigue. Int. J. Sports Med..

[27] Ferreira J.C., Rolim N.P., Bartholomeu J.B., Gobatto C.A., Kokubun E., Brum P.C. (2007). Maximal lactate steady state in running mice: Effect of exercise training. Clin. Exp. Pharmacol. Physiol..

[28] Duarte J.M., Agostinho P.M., Carvalho R.A., Cunha R.A. (2012). Caffeine consumption prevents diabetes-induced memory impairment and synaptotoxicity in the hippocampus of NONcZNO10/LTJ mice. PLoS ONE.

[29] Lalonde R. (2002). The neurobiological basis of spontaneous alternation. Neurosci. Biobehav. Rev..

[30] Sarnyai Z., Sibille E.L., Pavlides C., Fenster R.J., McEwen B.S., Toth M. (2000). Impaired hippocampal-dependent learning and functional abnormalities in the hippocampus in mice lacking serotonin (1A) receptors. Proc. Natl. Acad. Sci. USA.

[31] Kraeuter A.K., Guest P.C., Sarnyai Z. (2019). The Y-Maze for Assessment of Spatial Working and Reference Memory in Mice. Pre-Clin. Models..

[32] Bae J.Y. (2018). Aerobic Exercise Increases Meteorin-Like Protein in Muscle and Adipose Tissue of Chronic High-Fat Diet-Induced Obese Mice. Biomed Res. Int..

[33] Dias K.A., Coombes J.S., Green D.J., Gomersall S.R., Keating S.E., Tjonna A.E., Hollekim-Strand S.M., Hosseini M.S., Ro T.B., Haram M. (2016). Effects of exercise intensity and nutrition advice on myocardial function in obese children and adolescents: A multicentre randomised controlled trial study protocol. BMJ Open..

[34] Frühbeck G., Méndez-Giménez L., Fernández-Formoso J.A., Fernández S., Rodríguez A. (2014). Regulation of adipocyte lipolysis. Nutr. Res. Rev..

[35] Bae J.Y., Woo J., Kang S., Shin K.O. (2018). Effects of detraining and retraining on muscle energy-sensing network and meteorin-like levels in obese mice. Lipids Health Dis..

[36] Duncan R.E., Ahmadian M., Jaworski K., Sarkadi-Nagy E., Sul H.S. (2007). Regulation of lipolysis in adipocytes. Annu. Rev. Nutr..

[37] Knechtle B. (2002). Exercise intensity and fat burning--theoretical principles and practical considerations. Praxis.

[38] Friedlander A.L., Casazza G.A., Horning M.A., Usaj A., Brooks G.A. (1999). Endurance training increases fatty acid turnover, but not fat oxidation, in young men. J. Appl. Physiol..

[39] Lennox R., Moffett R.C., Porter D.W., Irwin N., Gault V.A., Flatt P.R. (2015). Effects of glucose-dependent insulinotropic polypeptide receptor knockout and a high-fat diet on cognitive function and hippocampal gene expression in mice. Mol. Med. Rep..

[40] Zafonte R.D., Shih S.L., Iaccarino M.A., Tan C.O. (2018). Neurologic benefits of sports and exercise. Handb. Clin. Neurol..

[41] Fordyce D.E., Farrar R.P. (1991). Enhancement of spatial learning in F344 rats by physical activity and related learning-associated alterations in hippocampal and cortical cholinergic functioning. Behav. Brain Res..

[42] Van Praag H. (2008). Neurogenesis and exercise: Past and future directions. Neuromolecular Med..

[43] Sun L.N., Li X.L., Wang F., Zhang J., Wang D.D., Yuan L., Wu M.N., Wang Z.J., Qi J.S. (2017). High-intensity treadmill running impairs cognitive behavior and hippocampal synaptic plasticity of rats via activation of inflammatory response. J. Neurosci. Res..

[44] Baker J.S., Bailey D.M., Hullin D., Young I., Davies B. (2004). Metabolic implications of resistive force selection for oxidative stress and markers of muscle damage during 30 s of high-intensity exercise. Eur. J. Appl. Physiol..

